# Primary percutaneous coronary angioplasty and therapeutic hypothermia in out-of-hospital cardiac arrest

**DOI:** 10.1186/cc12247

**Published:** 2013-03-19

**Authors:** R Hunt, M Holl, A Bailey, P Macnaughton

**Affiliations:** 1 Derriford Hospital, Plymouth, UK

## Introduction

The benefit of primary percutaneous intervention (PCI) in the management of out-of-hospital cardiac arrest (OHCA) is not clear cut [1]. It has historically been used in patients with ST elevation on post-resuscitation electrocardiogram (ECG) although this is a poor predictor of acute coronary occlusion after cardiac arrest [2]. This study investigates the benefit of PCI regardless of post-resuscitation ECG. Benefit is widely claimed for therapeutic hypothermia, so cooling parameters were included.

## Methods

We analysed all 41 consecutive adults admitted post OHCA to a university hospital ICU between January 2010 and December 2011. Patients received PCI regardless of ECG changes. A Cox proportional hazards model was used to determine the relationship between PCI, cooling and survival to discharge. Routinely collected data such as demographics and details of resuscitation (OHCA Utstein data) were also included.

## Results

**Figure 1:**
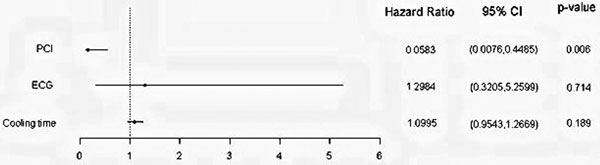
**Comparison of hazards ratios post Cox analysis**.

Survival to hospital discharge was 41% with 29% of survivors discharged to a neurological rehabilitation centre. Multivariate analysis using a Cox proportional hazards model showed PCI to be an independent predictive factor of survival, unrelated to ECG (hazards ratio, 0.0583; 95% CI, 0.0076 to 0.4485). Cooling had no significant impact on patient survival. See Figure 1.

## Conclusion

In this small retrospective study primary PCI appears to be an independent predictor of survival after OHCA. This is consistent with other studies suggesting benefit for primary PCI regardless of the post-resuscitation ECG [3]. Cooling was not found to improve survival to discharge but further analysis is required to determine impact on neurological function.
